# Construction of a recombinant bacmid DNA in order to express Neuraminidase in insect cell line

**DOI:** 10.1186/1753-6561-5-S1-P7

**Published:** 2011-01-10

**Authors:** S Najafi, F Behzadian, F Fotouhi, JF Mehrabadi, S Hossain Zadeh

**Affiliations:** 1 Department of Microbiology, Faculty of Biological Sciences, Islamic Azad University, Zanjan, Iran; 2 Department of Virology, School of Medical Sciences, Tarbiat Modares University, Tehran, Iran; 3 Influenza Unit, Department of Virology, Pasteur Institute of Iran, Tehran, Iran; 4 MARS Bioinformatics Institute, Tehran, Iran; 5 Department of Cellular and Molecular Biology, Faculty of Biological Sciences, Science and Research Branch of Islamic Azad University, Tehran, Iran

## 

Non-replicating virus-like particles (VLPs) have been suggested as a promising platform for viral vaccines. Recently several studies have introduced VLPs produced in insect cells as a vaccine candidate against influenza infection. Neuraminidase (NA), one of the two membrane glycoproteins of influenza A viruses has been employed to construct such influenza VLPs to induce protective immunity.

In this study, the human influenza virus (A /New Caledonia 20/1999/ (H1N1)) was propagated in MDCK cell culture. Viral RNA was extracted using RNX-plus solution. Complementary DNA synthesis was carried out using uni-12 primer. NA open reading frame (1413 bp) was amplified by RT-PCR using high fidelity Taq DNA polymerase. The amplicon was purified from the gel and cloned into pFastBac1 plasmid through SalI/XhoI sites. After verification of cloned NA by restriction analysis, it was subjected to automated sequencing bidirectionally. The recombinant pFastBacNA vector was transformed to E.coli DH10Bac cells which harbor bacmid DNA and helper plasmid to create NA recombinant bacmid. Restriction map analysis and sequencing results confirmed the fidelity of NA sequence. Homologous recombination between pFastBacNA and bacmid and creation of NA recombinant bacmid was verified by PCR using NA specific and M13 universal primers.

In an ongoing study, the cultured Sf9 (Spodoptera frugiperda) insect cell line will be transfected with NA recombinant bacmid to produce recombinant baculovirus expressing NA gene. This production is in its native highly glycosylated form appropriate to use in vaccine research projects. Recombinant baculovirus expressing NA gene can be also used with other individual recombinant baculoviruses expressing HA and M1 genes in production of influenza VLPs.

